# Mutations Related to Antiretroviral Resistance Identified by Ultra-Deep Sequencing in HIV-1 Infected Children under Structured Interruptions of HAART

**DOI:** 10.1371/journal.pone.0147591

**Published:** 2016-01-25

**Authors:** Jose Manuel Vazquez-Guillen, Gerardo C. Palacios-Saucedo, Lydia G. Rivera-Morales, Jorge Garcia-Campos, Rocio Ortiz-Lopez, Marc Noguera-Julian, Roger Paredes, Herlinda J. Vielma-Ramirez, Teresa J. Ramirez, Marcelino Chavez-Garcia, Paulo Lopez-Guillen, Evangelina Briones-Lara, Luz M. Sanchez-Sanchez, Carlos A. Vazquez-Martinez, Cristina Rodriguez-Padilla

**Affiliations:** 1 Laboratorio de Inmunología y Virología, Facultad de Ciencias Biológicas, Universidad Autónoma de Nuevo León, Monterrey, Nuevo León, México; 2 División de Investigación, Departamento de Pediatría e Infectología Pediátrica, Unidad Médica de Alta Especialidad, Hospital de Especialidades No. 25, Instituto Mexicano del Seguro Social, Monterrey, Nuevo León, México; 3 Departamento de Bioquímica y Medicina Molecular, Facultad de Medicina and Centro de Investigación y Desarrollo en Ciencias de la Salud, Universidad Autónoma de Nuevo León, Monterrey, Nuevo León, México; 4 Institut de Recerca de la SIDA, IrsiCaixa, Hospital Universitari Germans Trias i Pujol, Universitat Autònoma de Barcelona, Badalona, España and Cátedra de la SIDA, Universitat de Vic, Vic, España; 5 Laboratorio Estatal de Salud Pública del Estado de Nuevo León, Secretaría de Salud, Nuevo León, México; 6 Unidad de Infectología “Dr. Juan I. Menchaca”, Hospital General Regional No. 45, Instituto Mexicano del Seguro Social, Jalisco, México; University of British Columbia, CANADA

## Abstract

Although Structured Treatment Interruptions (STI) are currently not considered an alternative strategy for antiretroviral treatment, their true benefits and limitations have not been fully established. Some studies suggest the possibility of improving the quality of life of patients with this strategy; however, the information that has been obtained corresponds mostly to studies conducted in adults, with a lack of knowledge about its impact on children. Furthermore, mutations associated with antiretroviral resistance could be selected due to sub-therapeutic levels of HAART at each interruption period. Genotyping methods to determine the resistance profiles of the infecting viruses have become increasingly important for the management of patients under STI, thus low-abundance antiretroviral drug-resistant mutations (DRM’s) at levels under limit of detection of conventional genotyping (<20% of quasispecies) could increase the risk of virologic failure. In this work, we analyzed the protease and reverse transcriptase regions of the *pol* gene by ultra-deep sequencing in pediatric patients under STI with the aim of determining the presence of high- and low-abundance DRM’s in the viral rebounds generated by the STI. High-abundance mutations in protease and high- and low-abundance mutations in reverse transcriptase were detected but no one of these are directly associated with resistance to antiretroviral drugs. The results could suggest that the evaluated STI program is virologically safe, but strict and carefully planned studies, with greater numbers of patients and interruption/restart cycles, are still needed to evaluate the selection of DRM’s during STI.

## Introduction

Highly Active Antiretroviral Therapy (HAART) has been a significant advance for the treatment of HIV infection; however, problems that emerge during its administration, such as chronic toxicity, poor adherence and costs, are factors contributing to treatment failure and limiting its long-term success. Recently, evaluation of Structured Treatment Interruptions (STI) has been proposed as an alternative therapeutic strategy to reduce complications associated with continuous HAART administration [1]. Based on the establishment of treatment periods followed by drug-free periods long enough to allow significant viral rebound but with a controlled maintenance of the viral replication [2], STI could induce an increase of the HIV-specific immune response and, consequently, progressively smaller viral rebounds [3, 4]. However, information available about STI in pediatric patients remains limited and such information in adults is still controversial. The emergence of drug-resistance continues to be a major risk for the STI strategy and it is suspected that low serum levels of antiretroviral drugs during the viral rebounds could be selecting for drug-resistant HIV variants [5, 6]. Mutations associated with drug resistance are usually detected through standard sequencing; however, the quasispecies nature of HIV-1 complicates the detection of low-abundance drug-resistant mutations (DRM’s) [7, 8]. Quasispecies carrying DRM’s at levels <20% of the viral population can outgrow under selective pressure exerted by sub-therapeutic levels of the drug, becoming the major virus population and subsequently leading to therapy failure [9, 10]. The aim of this study was to evaluate the presence of both low-abundance and high-abundance (≥20% of viral quasispecies) DRM’s by ultra-deep sequencing in viral rebounds of two HIV-1-infected pediatric patients who underwent a STI program of HAART.

## Materials and Methods

### Patients and STI program

Two HIV-1 infected children with a viral load (VL) undetectable by HAART (<400 copies/ml), without immunosuppression according to the 1994 CDC classification [11] and HIV asymptomatic for at least the last twelve months were submitted to a STI program. HAART was interrupted for 4 weeks followed by 12 weeks on treatment, until completion of three interruption/restart cycles (4 weeks off/12 weeks on) as previously described [4, 6]. The VL, CD4+ and CD8+ cell counts as well as the clinical status of the patients were evaluated after each interruption period and at 6 and 12 weeks after HAART was restarted. VL was assessed using the Cobas Amplicor HIV-1 Monitor test, version 1.5 (Roche Diagnostics, Branchburg, NJ, USA), the detection limit of which is 400 copies/ml. Lymphocyte counts were measured by standard flow cytometry using the BD Standard Simultest / IMK Lymphocyte Kit (BD Biosciences, San Jose, CA, USA). The study was approved by the research ethics committee at the Hospital de Especialidades No. 25 of the Instituto Mexicano del Seguro Social and written informed consent from the parents or guardians of each patient was obtained.

### Amplicon generation and preparation of library for ultra-deep sequencing

Viral RNA was isolated from plasma samples collected at the end of each HAART interruption period (viral rebound) using the MagMax Viral RNA Isolation kit (Ambion, Foster City, CA, USA). Reverse transcription was performed with Super Script III First-Strand Synthesis System (Invitrogen, Carlsbad, CA, USA) and random hexamer primers according to manufacturer’s instructions. Using the HXB2 reference sequence (GenBank K03455.1), a set of five pairs of primers was designed to amplify overlapping regions of the protease and the first 1,348 nucleotides of the reverse transcriptase. Each one consisted of the Universal Sequence 454, A or B, at its 5' end required for labeling the amplicons of each sample prior to the emulsion-PCR reaction (emPCR) and the complementary region to the HIV-1 sequence at the 3' end (Table 1). The amplification reactions were performed independently with the cDNA from each sample as template using the Platinum PCR SuperMix High Fidelity (Invitrogen, Carlsbad, CA, USA). Temperatures in the thermocycler were 94°C for 2 min, followed by 40 cycles of 94°C for 15 sec, 54–58°C for 20 sec and 68°C for 1 min. In addition, a final extension period of 68°C for 4 min was programmed. All amplicons were analyzed using the Agilent DNA chips 12,000 Kit (Agilent Technologies, Santa Clara, CA, USA) to assess their approximate concentration, size and purity. Equimolar mixtures of the five amplicons for each viral rebound sample were prepared. Subsequently, for the removal of oligonucleotide dimers we used the Pippin Prep (Sage Science, Beverly, MA, USA) with 2% agarose gels, and fragments over 400 bp were recovered through electroelution. Amplicons were purified with 1.8 volumes of Agentcurt Ampure XP (Beckman Coulter, Beverly, MA, USA) and evaluated with the Agilent DNA chip 12,000. Each amplicon mixture was processed in a second amplification reaction to add the fusion primers containing the Adapter sequence from Roche-454, the molecular identifier (MID) tags in both sense and anti-sense primers and 454-Universal A/B Sequence at its 3’ end (Integrated DNA Technologies Inc., Coralville, IA, USA) (Table 2). The temperatures programmed in the thermocycler were 94°C for 1 min and 6 cycles of 94°C for 30 sec, 55°C for 30 sec and 68°C for 1 min, with a final extension step at 68°C for 2 min. Products from second amplification, containing the five labeled amplicons for each sample, were purified with 1.8 volumes Ampure XP Agentcurt, evaluated with DNA Agilent 12,000 chips and quantified using Quant-iT PicoGreen dsDNA Assay Kit (Invitrogen). Finally, an equimolar mixture of the labeled amplicons was made to generate a library in stock concentration of 1 x 10^9^ molecules/μL and a working dilution of 1 x 10^7^ molecules/μL was prepared. For the emPCR, a 0.66:1 ratio of library molecules was mixed with DNA capture beads following the emPCR Lib-A protocol from Roche (Roche Company 454 Life Sciences, Branford, USA). The enriched DNA-capture beads were sequenced in GS Junior Titanium Series equipment (Roche) following manufacturer's instructions.

**Table 1:** Primers with 454-Adaptors (A and B) used to amplify the overlapping protease- and reverse-transcriptase coding regions.

Amplicon	Primer Name	UNIV-A / UNIV-B + HXB2 Complementary Sequence^a^	HXB2 Position	Tm (°C)^b^
1^st^	Par1F	GTAAAACGACGGCCAGcccaccagaagagagcttca	2160–2180	60.5
	Par1R	CAGGAAACAGCTATGACtttaacttttgggccatcca	2595–2615	60.3
2^nd^	Par2F	GTAAAACGACGGCCAGtagggggaattggaggtttt	2392–2412	59.6
	Par2R	CAGGAAACAGCTATGACtgcatcacccacatccagta	2872–2891	61.0
3^rd^	Par3F	GTAAAACGACGGCCAGggcctgaaaatccatacaatac	2701–2722	57.5
	Par3R	CAGGAAACAGCTATGACgccctatttctaagtcagatccta	3115–3138	57.3
4^th^	Par4F	GTAAAACGACGGCCAGcacagggatggaaaggatca	2998–3017	60.9
	Par4R	CAGGAAACAGCTATGACtgcccctgcttctgtatttc	3531–3550	60.2
5^th^	Par5F	GTAAAACGACGGCCAGtccttagaggaaccaaagca	3394–3413	57.5
	Par5R	CAGGAAACAGCTATGACcctgttagctgccccatct	3875–3893	60.2

^a^ Upper case letters indicates the Roche 454-Universal Sequence A or B, and lower case letters the complementary region to HXB2 reference sequence.

^b^ The Tm corresponds only for the complementary sequence with the HXB2 reference.

**Table 2:** Complete sequence of the 454-fusion primers used for the second amplification in library preparation for ultra-deep sequencing.

Patient (STI)	MID^a^	Name	Complete Sequence (5' > 3')			
			Adaptor A / B	Key	MID	Univ-A / Univ-B
1 (1^st^)	GS-MID03	Adapter-MID03-U-F	cgtatcgcctccctcgcgcca	tcag	agacgcactc	gtaaaacgacggccag
		Adapter-MID03-U-R	ctatgcgccttgccagcccgc	tcag	agacgcactc	caggaaacagctatgac
1 (2^nd^)	GS-MID04	Adapter-MID04-U-F	cgtatcgcctccctcgcgcca	tcag	agcactgtag	gtaaaacgacggccag
		Adapter-MID04-U-R	ctatgcgccttgccagcccgc	tcag	agcactgtag	caggaaacagctatgac
1 (3^rd^)	GS-MID05	Adapter-MID05-U-F	cgtatcgcctccctcgcgcca	tcag	atcagacacg	gtaaaacgacggccag
		Adapter-MID05-U-R	ctatgcgccttgccagcccgc	tcag	atcagacacg	caggaaacagctatgac
2 (1^st^)	GS-MID08	Adapter-MID08-U-F	cgtatcgcctccctcgcgcca	tcag	ctcgcgtgtc	gtaaaacgacggccag
		Adapter-MID08-U-R	ctatgcgccttgccagcccgc	tcag	ctcgcgtgtc	caggaaacagctatgac
2 (2^nd^)	GS-MID09	Adapter-MID09-U-F	cgtatcgcctccctcgcgcca	tcag	tagtatcagc	gtaaaacgacggccag
		Adapter-MID09-U-R	ctatgcgccttgccagcccgc	tcag	tagtatcagc	caggaaacagctatgac
2 (3^rd^)	GS-MID10	Adapter-MID10-U-F	cgtatcgcctccctcgcgcca	tcag	tctctatgcg	gtaaaacgacggccag
		Adapter-MID10-U-R	ctatgcgccttgccagcccgc	tcag	tctctatgcg	caggaaacagctatgac

^a^ Molecular identifier tag from Roche 454 Amplicon Fusion Primer Design.

### Sequencing Analysis

The sequencing analysis was made using the GS-Amplicon Variant Analyzer (AVA) software (v 2.8.0). Sequences were demultiplexed using either 5’ or 3’ MID barcodes. AVA was then used to generate alignments based on error corrected consensus reads for each sample. An in-house perl code was used to call variants at the codon level. A minimal coverage of 500 reads per position was required for further analysis to ensure maximal probability of detecting low-abundance DRM’s. In addition, according to sequencing strand-dependent homopolymer related error patterns and routine negative control results, only those variants showing frequency values on forward and reverse reads within a 1 Log ratio and an overall frequency greater than 1.0% were considered true variants and used for downstream analysis. A variant list containing all drug resistant mutations was reported in the Stanford HIV Drug Resistance Database algorithm (HIVdb, version 6.3.1, updated 09/20/13) through the HIVdb Sierra interface.

### Definition of low-abundance and high-abundance drug resistance mutations

For the purpose of discussion in this paper, we used the term “low-abundance” DRM’s to define mutation that can be detected at <20% of viral quasispecies and “high-abundance” DRM’s as mutations detected at ≥20% of viral quasispecies through ultra-deep sequencing [8].

## Results

### Patient characteristics

Two children were included in the study. Patient No. 1 was a 13.9 year-old female with immune category B3. Patient No. 2 was a 15 year-old male with immune category B2. Both patients acquired HIV-1 infection perinatally and they were receiving zidovudine (AZT) + lamivudine (3TC) plus ritonavir (RTV) as a HAART regimen at the recommended pediatric dosages. Full-dose RTV as the only protease inhibitor (PI) was still recommended for the initial HAART regimen in children in Mexico at the time this study began (Table 3).

**Table 3:** Baseline characteristics of two HIV-1 infected children included in a structured treatment interruption (STI) program of HAART.

	Patient No. 1	Patient No. 2
Age (years)	13.9	15
Sex	Female	Male
Previous ART (months)	AZT+3TC+RTV (40)	AZT+3TC+RTV (40)
HAART regimen^a^ (months)	Combivir+RTV (48)	Combivir+Kaletra (38)
Viral load	<400 copies/mL	<400 copies/mL
	(<2.6 log_10_/mL)	(<2.6 log_10_/mL)
Clinical-immune category^b^	B3	B2
HIV-1 subtype^c^	B	B

ART, Antiretroviral treatment; HAART, Highly Active Antiretroviral Therapy; AZT, Zidovudine; RTV, Ritonavir; 3TC, Lamivudine.

^a^ At the beginning of the Structured Treatment Interruption program.

^b^ According to the 1994 Centers for Disease Control and Prevention (CDC) classification [11].

^c^ Determined by the computer program REGA HIV-1 Subtyping Tool and reported in a previous study [4].

### Response to the STI program

There were no symptoms related to HIV infection and no adverse events related to HAART were observed during the follow-up of the patients. In both patients, the third viral rebound was significantly lower than first and second. VL always fell under detection limits after the therapy was restarted. According to the absolute CD4+ T-Cell counts, Patient No. 1 experienced moderated immunosuppression at the second interruption period (361 cells/ml); however, subsequently counts of these cells tended to increase reaching 791 at the end of follow up. Patient No. 2 experienced severe immunosuppression at the first interruption period (147 cells/ml), but it was transitory reaching afterwards CD4+ T-Cell counts similar than baseline (Table 4).

**Table 4:** Viral load and T lymphocytes counts of HIV-1 infected children included in a structured treatment interruption program of HAART.

Patient	Time of follow-up											
	Months		Weeks									
	-12	-6	0	4	10	16	20	26	32	36	42	48
	HAART			1^st^ STI	HAART		2^nd^ STI	HAART		3^rd^ STI	HAART	
**No. 1**												
VL	<400	<400	<400	9730	<400	<400	10500	135	<400	2660	<400	<400
VL Log_10_	<2.6	<2.6	<2.6	3.99	<2.6	<2.6	4.02	2.13	<2.6	3.42	<2.6	<2.6
CD4+ %	51%	56%	53%	43%	35.3%	46.6%	20.3%	30.4%	25.6%	22.5%	24.2%	28.1%
Absolute CD4+	1157	1241	1255	412	719	749	361	895	807	465	549	791
Absolute CD8+	1368	1778	1561	997	987	1586	840	1161	1419	819	905	1157
**No. 2**												
VL	<400	<400	<400	52900	723	<400	88800	<400	<400	24200	<400	<400
VL Log_10_	<2.6	<2.6	2.08	4.72	2.86	<2.6	4.95	<2.6	<2.6	4.38	<2.6	<2.6
CD4+ %	61%	ND	35%	4.9%	33.8%	14.3%	24.2%	14.3%	26.3%	30.4%	31%	23%
Absolute CD4+	1022	923	560	147	723	345	536	345	478	493	528	473
Absolute CD8+	611	616	523	2055	600	1566	571	1566	669	599	624	735

HAART: Highly Active Antiretroviral Therapy; STI: Structured Treatment Interruption; VL: Viral Load; ND: Not determined.

### Sequencing analysis and drug resistance interpretation

Amplicons covering part of the nucleotide sequences of the *pol* gene, including the complete protease-coding region (residues 1–99) and part of the reverse transcriptase-coding region (residues 1–448) were analyzed in the viral rebounds generated in patients during interruption periods of the STI program. The dataset have been deposited in the NIH/SRA repository with the accession number PRJNA284433. Genotypes for resistance prediction were obtained using the HIVdb of the Stanford University [12]. No mutations for major or minor resistance to PI were found, and the analysis indicates susceptibility to all PI currently in clinical use. All mutations in the protease region that were detected corresponded to high-abundance DRM’s as they occurred in average frequency values of both forward and reverse coverage close to 100% of the reads. Additionally, mutations that are associated with resistance to nucleoside reverse transcriptase inhibitors (NRTI) or non-nucleoside reverse transcriptase inhibitors (NNRTI) were found. The mutation K101E was detected in the three viral rebounds of Patient No. 1 and V108I was detected only at the third viral rebound of this patient. These mutations are associated to NNRTI resistance and were found as low-abundant DRM’s (< 20%). In Patient No. 2, the E138A mutation was detected at high-abundance (> 99% of the reads) in the three viral rebounds. V90I was detected in the first viral rebound as a high-abundance DRM’s but in the second and third rebound it was detected as a low-abundance. V106I was detected only in the first viral rebound and A62V emerged at the third viral rebound of this patient. The A62V mutation is associated to NRTI resistance; all of the others found are related to NNRTI resistance (Table 5).

**Table 5:** Drug resistance interpretation of low-abundance and high-abundance mutations detected by ultra-deep sequencing in two HIV-1 infected children underwent a structured treatment interruption program of HAART.

Patient	STI	Class	Mutation	FreqAvg (%)^a^
No. 1	1^st^	PI	I93L	100
		PI	L63P	100
		PI	V77I	100
		NNRTI	K101E	1.86
	2^nd^	PI	I93L	100
		PI	L63P	100
		PI	V77I	99.94
	3^rd^	PI	I93L	100
		PI	L63P	100
		PI	V77I	99.90
		NNRTI	V108I	2.59
		NNRTI	K101E	1.01
No. 2	1^st^	PI	L63P	99.69
		PI	I64V	99.45
		NNRTI	E138A	100
		NNRTI	V106I	23.74
		NNRTI	V90I	93.74
	2^nd^	PI	I64V	100
		PI	L63P	99.34
		NNRTI	E138A	100
	3^rd^	PI	L63P	100
		PI	I64V	97.40
		NRTI	A62V	1.72
		NNRTI	E138A	99.72
		NNRTI	V90I	14.61

^a^ Frequency average in 90,000 reads.

^b^ HIVdb Genotyping resistance interpretation algorithm. STI, Structured Treatment Interruption; PI, Protease inhibitor; NRTI, Nucleoside Reverse Transcriptase Inhibitor; NNRTI, Non-Nucleoside Reverse Transcriptase Inhibitor.

## Discussion

Antiretroviral drugs represent a significant progress in the treatment of HIV infection; however, problems that emerge during their administration limit their long-term benefits [13]. In pediatric patients, the burden of chronic treatment is the most common reason for treatment interruption; however, other side effects, such as the development of physiological problems, are involved directly with treatment adherence [14, 15]. As a result, STI has been proposed as an alternative to continuous HAART, since they have the potential to reduce problems of chronic toxicity and adherence to treatment [4]. Because the progression of HIV-related illness is often faster in children than in adults, it has not been conclusively shown whether strategies based on the interruption of treatment may also work in the pediatric population, and therefore the information available for these patients is limited [2, 4, 16].

In a previous study by our research group [4], four children with HIV-1 infection under virological control were subjected to a scheme of STI in order to assess its immunological and clinical impact. The HAART of each child was interrupted for 4 weeks, followed by 12 weeks under treatment, until three interruption/restart cycles were completed. In all four patients, progressively lower viral rebounds were observed with a vigorous development of T CD8+ cells and an initial decrease in the response of T-helper cells, followed by a subsequent increase. In a second study, using a standard genotyping analysis and the HIVdb [12], the sequences codifying for the protease and reverse transcriptase were analyzed in samples corresponding to the viral rebounds of the STI program, finding no resistance to any of the PI or reverse transcriptase inhibitors commercially available [6]. Despite the limitations of the number of patients analyzed, the results of that previous work suggested that STI, in the context evaluated, could be safe; however, before suggesting new research in different populations of children, it is necessary to consider the genetic heterogeneity of the viral subpopulations during HIV infection and to assess the presence or absence of mutations associated with antiretroviral resistance even in minority viral populations infecting a patient. In effect, the natural tendency to errors of the reverse transcriptase, combined with the high viral replication activity, result in the generation of genetically related viral variations called quasispecies with important implications on the dynamics of the infection [17]. Moreover, one of the major limitations of standard genotyping tests is their inability to detect quasispecies that occur in less than 20–25% of the circulating viral population in a patient [10]. These subpopulations in low-abundance can lead to mutations associated with antiretroviral resistance and can develop rapidly to become predominant under conditions of selective pressure which eventually lead to therapeutic failure [18]. An alternative to identify these minority variants is the use of ultra-deep sequencing, also known as next-generation sequencing (NGS) technologies [19, 20, 21, 22]. The possibility of detecting mutations associated with resistance to antiretrovirals drugs of various viral quasispecies, including those of low-abundance is the main advantage of the ultra-deep sequencing over the traditional Sanger method. In this work, ultra-deep sequencing was used in a short amplicon design to assess the selection of mutations associated with antiretroviral resistance during viral rebounds of two children with chronically undetectable VL included in a STI schedule of 4 weeks off / 12 weeks on HAART.

The L63P, V77I, and I93L mutations in the protease region were found in patient No. 1 and L63P and I64V in patient No 2. The HIVdb genotypic resistance interpretation algorithm classifies these mutations as “other mutations” and refers them as accessory mutations. Such mutations are not associated with antiretroviral resistance. The L63P mutation is a common polymorphism in patients receiving PI; V77I is a common polymorphism associated with treatment with Nelfinavir (NFV) and the I93L mutation is also a common polymorphism associated with PI therapy in subtype B viruses. The HIVdb does not show comment for the I64V mutation. However, resistance mutations in the protease gene are classified as “major” or “minor” in the 2014 Update of the Drug Resistance Mutation in HIV-1 from the International Antiviral Society-USA (IAS-USA) [23]. According to the IAS-USA list, all the mutations in protease region of our patients are defined as minor resistance mutations. L63P is associated with Lopinavir (LPV) minor resistance; V77I with NFV, Indinavir (IDV), and Saquinavir (SQV) minor resistance; I93L and I64L are related to minor resistance against Atazanavir (ATV). The minor mutations do not have a substantial effect on the phenotype and often are generally considered to be accessory mutations but they may improve replication of viruses containing major mutations [23]. Comparing viral rebounds in each patient, it was observed that substitutions in the protease-coding region were the same in each rebound, it may thus be suggested that selection of new mutations was not induced during the three periods of interruption/resumption. Instead, mutations detected in the protease may have been present before the STI scheme was initiated although it is hard to make correct interpretations of the data without a baseline sequence prior to the STI program. These mutations were also found in the previous study using standard genotyping to assess the effect of the STI [6]. Additionally, another study also reported the presence of these changes in naïve patients [24]. In the present work, the mutations in the protease were always detected as a high-abundance and variation in their frequency never was shown (Table 5). These observations may suggest that the STI program, as planned in this study, could be safe by not selecting mutations associated with resistance to PI.

On the other hand, several mutations associated with low-level or intermediate-level resistance to NNRTI were found in the viral rebounds of both patients. In two viral rebounds of patient No. 1, the K101E mutation was found, which causes intermediate resistance to Nevirapine (NVP) and low-level resistance to Efavirenz (EFV) and Etravirine (ETR) and, in combination with M184I, causes intermediate resistance to Rilpivirine (RPV). The mutation was maintained at low-abundance and it never surpassed 2% of the reads, despite the cycles of treatment interruption/restart in this patient. In the third rebound of the same patient, the V108I mutation was detected, which is selected by NVP, EFV and ETR and may slightly reduce the susceptibility to NVP and EFV. The E138A mutation detected in the three viral rebounds of patient No. 2 is a common polymorphic change, which can be selected for the treatment with ETR and RPV. This mutation may slightly decrease susceptibility to ETR and RPV. The V90I substitution, found in the first and third viral rebounds of patient No. 2 is a common accessory polymorphism that may be slightly selected for the treatment with NNRTI *in vitro* and/or *in vivo* and is associated with minimal or no reduction in the susceptibility to ETR. In the first rebound of this patient, the V106I mutation was detected, which is a polymorphic mutation selected by the EFV therapy and which, in combination with V179D, can reduce susceptibility to NVP and EFV. The A62V mutation, found in the third rebound of patient No. 2, is the only one associated with NRTI resistance but it requires the presence of the changes K65R and Q151M or the T69 insertion to reduce susceptibility to NRTI. Alone, A62V has no effect on the susceptibility to antiretroviral drugs, and although it is indicated as a resistance mutation to NRTI, the viruses that present it are susceptible to all antiretroviral drugs.

Since mutations K101E in patient No. 1 and E138A and V90I in patient No. 2 were present from the first period of interruption. It is thus possible that these mutations were not generated during the interruption/restart periods of HAART, but they were already present before patients be included in the study; however, no genotyping study was carried out prior to initiating the STI program to ensure this. In addition, V108I in patient No. 1 and A62V in patient No. 2 emerged as low-abundance DRM’s at the third viral rebound of the STI program, so subsequent evaluations are needed to monitoring the behavior of these mutations. Importantly, the mutations related with resistance to NNRTI detected are generally selected by the treatment with the same NNRTI and both children in our study never received and were not receiving such antiretroviral drugs.

In the present study, not any HIV-infection related symptom or HAART related side event was identified, such as we observed in our previous study that included four pediatric patients submitted to the same STI program [4]. Similarly, CD4+ lymphocyte counts fell during periods of treatment interruption but subsequently recovered, and although they not reached baseline levels at final follow-up these counts tended to increasing. Nonetheless, these decreasing in CD4+ lymphocyte counts may cause concerns about the possibility of subsequent development of more immune problems; additional interruption-restart periods were necessary to demonstrate whether these counts rebounded back to levels prior to STI. Because only one event of severe immunosuppression accordingly to the absolute CD4+ T-Cell counts occurred, which was transitory, these results suggest that the risk of loss of control of the immune response with the subsequent development of secondary clinical events is low [3, 4].

In summary, it could be concluded that the STI program did not lead to the development of any meaningful DRM’s which suggests that the detected mutations do not represent a risk for virologic failure during subsequent therapy in patients. Thus, this study highlight the need to continue studies about the impact of STI in pediatric patients with a strict and careful planning and with a greater number of interruption/restart cycles to evaluate the behavior of DRM’s that can emerge.

## Ethical Approval

This study was approved by the institutional research and ethics review board of the Hospital de Especialidades No. 25, Instituto Mexicano del Seguro Social, Monterrey, Mexico.
